# Clinical trials of neoadjuvant immune checkpoint inhibitors for early-stage operable colon and rectal cancer

**DOI:** 10.1007/s00262-023-03480-w

**Published:** 2023-08-01

**Authors:** Torhild Veen, Arezo Kanani, Dordi Lea, Kjetil Søreide

**Affiliations:** 1 Department of Gastrointestinal Surgery, Stavanger University Hospital, Stavanger, Norway; 2 Gastrointestinal Translational Research Unit, Laboratory for Molecular Medicine, Stavanger University Hospital, Stavanger, Norway; 3 Department of Pathology, Stavanger University Hospital, Stavanger, Norway; 4 Department of Clinical Medicine, University of Bergen, Bergen, Norway

**Keywords:** Immunotherapy, Immune checkpoint inhibitor, Mismatch repair deficiency, Colon cancer, Rectal cancer, Study design

## Abstract

**Background:**

Immune checkpoint inhibitors (ICI) have become first-line treatment for metastatic colorectal cancer (CRC) with deficient mismatch repair (dMMR). Despite the remarkable response reported in preliminary trials, the role of ICI in patients with early-stage, operable CRC remains unclear. The aim of this study was to investigate trials on neoadjuvant ICI in operable CRC.

**Materials and methods:**

Scoping review of clinical trial registries (Clinicaltrials.gov and EU clinical trial registers) and PubMed/Medline database of trials on neoadjuvant ICI for operable CRC was done up to December 2022.

**Results:**

Some 40 trials investigating neoadjuvant ICI for early-stage, operable CRC were identified, including five published trials and three conference abstracts. Preclinical phase I/II trial predominated with only three clinical phase III trials. Few trials investigated neoadjuvant ICI as the only intervention (monotherapy). Trials in rectal cancer were designed for combined ICI with chemo(radio)therapy, only 8 trials stating an MSI/dMMR status for inclusion, one designed for MSS/pMMR only and, the rest agnostic for MMR status. Thirty-eight (95%) trials investigated programmed cell death protein 1 (PD-1) or programmed cell death ligand 1 (PD-L1) inhibitors. PD-1/PD-L1 inhibitors were combined with vascular endothelial growth factor (VEGF) inhibitor or with cytotoxic T-lymphocyte-associated protein-4 (CTLA-4) inhibitor, in two trials each, respectively. Pathological complete response as primary outcome after surgery was the most frequently used study endpoint. In rectal cancer, six trials included a “watch and wait” strategy for patients with complete clinical response. No “watch and wait” study design for colon cancer after neoadjuvant ICI were identified.

**Conclusion:**

High response rates from neoadjuvant ICI in early-stage colon and rectal cancer are reported in phase I/II studies. Contemporary trial designs are heterogeneous, with few comparable inclusion criteria, use of several drug combinations and durations and, wide variation of endpoints reported. Harmonizing clinical and translational aspects including survival data is needed for improved future trial designs with clinical impact.

**Supplementary Information:**

The online version contains supplementary material available at 10.1007/s00262-023-03480-w.

## Introduction

Immune checkpoint inhibitors (ICI) are approved as first-line therapy for the treatment for unresectable, metastatic colorectal cancer (CRC) with mismatch repair deficiency (dMMR) [1]. Of note, patients with cancers harboring dMMR are expected to occur at a much higher rate in both operable colon and rectal cancer, but currently no immunotherapy is approved for early-stage disease. While less than 5% of patients with metastatic CRC have dMMR [2, 3], up to 15–25% of patients with primary, early-stage (i.e. stage I–III; non-metastatic) CRC have tumors with dMMR or microsatellite instability (MSI) in the genome [2, 4]. Notably, up to 75–80% of patients with CRC present in an operable stage at time of diagnosis [5], and being offered surgery with curative intent. Preliminary clinical studies of neoadjuvant ICI have demonstrated very high clinical or pathological response rates in operable colon and rectal cancer [6–8], potentially suggesting that surgery may be deferred altogether in some patients. Hence, the preoperative use of ICI has the potential to radically change current management or, the oncological outcome, of select patients with operable colon or rectal cancers.

Globally, a formidable challenge is presenting with the unexplained increase in early-onset colorectal cancers [9]. While some present early on a germline-mutation background, most are sporadic [10] with the sharpest increase seen in patients < 50 years of age. Also, screening-programs may increase the rate of early-stage disease in a younger population further. For young patients with advanced or locally invasive tumors and who may be eligible for immunotherapy, such a neoadjuvant strategy with immune checkpoint inhibitors may help facilitate down-staging of large tumors, allow for more organ-sparing surgery and improved functional outcomes, even with a complete non-operative treatment in those with a complete response.

Notably, the prevalence of dMMR in early-stage CRC is high, with a predilection for right-sided cancers which are found to be more ‘immune hot’ by the prevalence of tumor-infiltrating lymphocytes [11]. Further, treatment of early-stage disease represents an opportunity for disease management for when cure is the clear goal, yet morbidity should be minimized when possible. With current, conventional therapy about one-third of young patients treated for locally advanced rectal cancer experience long-term detrimental outcomes in function [12]. Also, treatment-related effects should aim for optimal long-term quality of life and maintenance of organ-function, particularly in the younger population with anticipated greater longevity.

Despite the very promising early results of ICI, further data is needed for patient selection, durability and evaluation of effects and long-term oncological outcomes in early-stage disease. Also, increased interest in ICI for tumors with proficient mismatch repair has emerged since a response is elicited in up to one-third of patients with pMMR tumors [13]. Thus, this paper summarizes the latest clinical data on published and emerging trials regarding ICI in early-stage operable colon and rectal cancer.

### Search strategy and selection criteria

References for this Review were identified through searches of PubMed and clinical trial registries with the medical subject heading (MeSH) search terms: “Colorectal cancer AND Immunotherapy”, “Colon cancer AND Immunotherapy” and “Rectal cancer AND Immunotherapy” from 2014 (FDA approval of pembrolizumab in September, 2014).

We explored www.ClinicalTrials.gov and the EU ClinicalTrials Register with search phrases “Colorectal cancer AND Immunotherapy”, “Colon cancer AND Immunotherapy” and “Rectal cancer AND Immunotherapy”. The available English literature in PubMed/Medline was searched using the same medical subject heading (MeSH) terms, alone or in combinations, to identify trials, study protocols and abstracts. A Google search was performed for conference abstracts, with focus on the ASCO and ESMO conferences, for the most recent years (2020 onwards).

Only studies reporting on use of ICI in a neoadjuvant setting for primary resectable or, locally advanced/potentially resectable, CRC were considered. All trials were assessed for study characteristics, including (but not limited to) clinical trial stage (phase I, II, III, IV); reported selection criteria, such as location in the large bowel (colon, rectum, or both); clinical stage at inclusion; whether proficient or deficient MMR (pMMR/dMMR) or microsatellite stable or instable (MSS/MSI) tumors were selected for; type of drug, combinations and duration and, endpoints stated for the studies.

Articles identified as editorials, letters, case reports or case series were excluded. Reviews were scrutinized for further referenced studies, as were reference lists of identified papers. Data from unresectable stage IV or studies on palliative effect of ICI in metastatic CRC were excluded. Abstracted data found in grey literature/conference abstracts were excluded if subsequent publications were found.

The most recent 5-year period was chosen to focus solely on the most updated clinical studies and clinical data. Tables 1 and 2 are generated by searching for clinical trials in registries for ongoing, active or, recruiting up to December 30th, 2022. All human clinical trials published in English with a full journal text available were reviewed. Articles were also identified through searches of the authors’ own files. Only papers published in English were reviewed. The final reference list was generated based on originality and relevance to the scope of this review.

**Table 1:** Neoadjuvant ICI trials in rectal cancer

NCT #/EudraCT (Study name)	Drug	Target	Mono/double therapy	*N*	Phase	Stage	MSI/MSS	End-point
NCT03854799 (AVANA)	Avelumab	PD-L1	Mono (+ CRT)	101	II	III	Both	pCR
NCT04357587	Pembrolizumab	PD-1	Mono (+ CRT)	10	I	III–IV	MSI/dMMR	AE, CSOT
NCT03127007 (R-IMMUNE)	Atezolizumab	PD-L1	Mono (+ CRT)	54	I/II	III	Both	AE, pCR
NCT04017455 (TARZAN)	Atezolizumab Bevacizumab	PD-L1 VEGF	Mono (+ RT)	38	II	I–III	Both	cCR
NCT04109755 (PEMREC)	Pembrolizumab	PD-1	Mono (+ RT)	25	II	III	MSS	TRR
NCT04130854 (INNATE)	AOX005M/Sotigalimab	Anti CD40	Mono (+ CRT)	58	II	II–III	Both	pCR
NCT04503694 (Regina trial)	Nivolumab	PD-1	Mono (VEGF + RT)	60	II	II/III	Both	pCR
NCT04518280 (TORCH)	Toripalimab	PD-1	Mono (+ CRT)	130	II	II/III	Both	pCR
NCT04558684	Camrelizumab	PD-1	Mono (+ CRT)	30	I/II	II/III	Both	cCR, DFS
NCT05176964	Tislelizumab	PD-1	Mono (+ CRT)	50	II	II/III	Both	pCR
NCT04621370 (PRIME-RT)	Durvalumab	PD-L1	Mono (+ CRT)	48	II	III	Both	pCR
NCT04231552	Camerelizumab	PD-1	Mono (+ CRT)	30	I/II	III	Both	pCR
NCT04643041 (BASKET)	N.s	PD-1	Mono	47	Not applicable	I–III	MSI/dMMR	1-yr DFS
NCT03921684	Nivolumab	PD-1	Mono (+ CRT)	29	II	III	Both	pCR
NCT04906044 (STARS-RC03)	Tislelizumab	PD-1	Mono (+ CRT)	30	I	I–III	Both	AE
NCT02921256 (NRG-GI002)	Pembrolizumab	PD-1	Mono (+ CRT)	362	II	II/III	Both	NAR score*
NCT04443543	Tislelizumab	PD-1	Mono (+ CRT)	222	II	II/III	MSI	cCR
NCT04751370	Nivolumab, Ipilimumab	PD-I/CTLA-4	Double (+ RT)	31	II	II/III	MSI/dMMR	pCR
NCT03503630	Compound 2,055,269	PD-L1	Mono (+ CRT)	44	II	II/III	Both	pCR
NCT02688712 (ExIST)	Galunisertib	TGFß-R1	Mono (+ CRT)	50	II	II–IV	Both	pCR
NCT02948348 (VOLTAGE)	Nivolumab, ipilimumab	PD-1 CTLA-4	Mono (+ CRT) Double (+ CRT)	90	I/II	III/IV	Both	pCR
NCT04928807 (UNION)	Camrelizumab	PD-1	Mono (+ CRT)	213	III	III	Both	pCR
NCT04663763	Sintilimab	PD-1	Mono (+ CRT)	40	II	II/III	Both	pCR
NCT04411537	PD-1-antibody	PD-1-	Mono (+ CRT)	50	II	II/III	MSS/pMMR	pCR
NCT04411524	PD-1-antibody	PD-1	Mono (+ CRT)	50	II	II/III	MSI/dMMR	pCR, DFS
NCT05507112	Tislelizumab	PD-1	Mono (+ CRT)	100	II	III	MSI/dMMR	pCR
NCT05484024 (STELLAR II)	Sintilimab (Tyvyt)	PD-1	Mono (+ CRT)	588	II/III	III	Both	pCR/cCR
NCT05479240	Tislelizumab	PD-1	Mono (+ RT)	96	II	II/III	Both	pCR
NCT05215379	Xintilimab (Tyvyt)	PD-1	Mono (+ CRT)	180	II/III	II/III	MSS	cCR at 6 mth (WW)
NCT04124601/2019–003865-17 (CHINOREC)	Ipilimumab nivolumab	CTLA-4 PD-1	Double (+ CRT)	80	II	II/III	Both	Safety, feasibility
NCT04165772	Dostarlimab	PD-1	Mono (+ CRT)	51	II	II/III	MSI/dMMR	pCR/cCR
NCT04293419 (DUREC trial)	Durvalumab	PD-1	Mono + TNT (CRT)	58	II	II/III	Both	pCR

*CRT* Chemoradiotherapy,*RT* Radiotherapy, *n.s.* “not specified”, *TGFß-R1* Tumor Growth Factor beta type 1 receptor, *pCR* pathological complete response, *AE* adverse events, *CSOT* completed surgical or oncological treatment, *cCR* clinical complete response, *TRR* Tumor regression rate, *DFS* disease free survival, *NAR score*, neoadjuvant rectal cancer score

**Table 2:** Neoadjuvant ICI trials in combined colon and rectal cancer

NCT #/EudraCT (Study name)	Drug	Target	Mono/double therapy	*N*	Phase	Stage	MSI/MSS	End-point
NCT03926338	Toripalimab	PD-1	Mono	100	I/II	I-III	MSI/dMMR	pCR
NCT04715633	Camrelizumab Apatinib	PD-1 VEGF	Double (+CRT or chemo only)	52	II	III	MSI/dMMR	cCR/pCR
NCT04895137 (BASKET II)	PD-1 monoclonal antibody	PD-1	Mono (+chemo)	42	II	III	MSS/pMMR	pCR
NCT05371197	Envafolimab	PD-L1	Mono	26	II	II/III	MSI/dMMR	pCR
NCT05197322 (NEOPRISM-CRC)	Pembrolizumab	PD-1	Mono	32	II	II/III	MSI/dMMR	pCR

*CRT* denotes Chemoradiotherapy, *RT* denotes Radiotherapy, *pCR* denotes pathological complete response, *cCR* denotes clinical complete response

## Results

The search (Supplementary Fig. 1) identified 40 trials on neoadjuvant ICI for resectable CRC in a non-metastatic and curative setting. These were reported to include patients with colon cancer in 4 trials, rectal cancer in 31 trials (Table 1) or, a combination of colon and rectal cancers as reported in Table 2.

### Immune checkpoint inhibitors in operable colon and rectal cancer

**Fig. 1:**
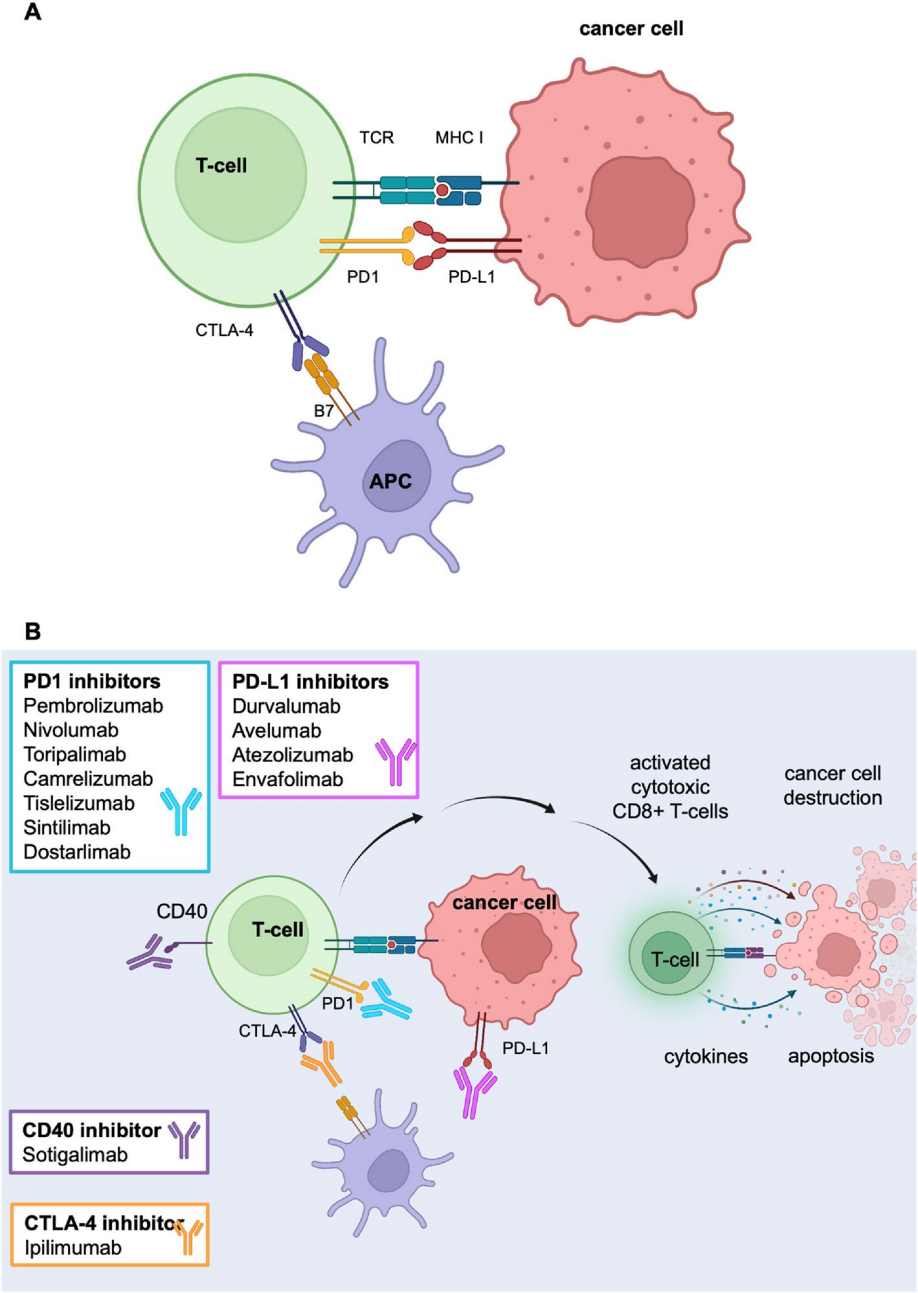
Simplistic overview of immune checkpoint inhibition and targets in neoadjuvant trials. **A** Inactive T-cell against cancer cell. Interaction between antigen-presenting cell (APC) and T-cell and cancer cell. T-cells are inactive due to binding of programmed death-1 and programmed death -ligand 1 (PD1 and PD-L1). Major histocompatibility complex (MHC)-T cell receptor (TCR)-dependent signaling demonstrates immune evasion by cancer cells expressing the inhibitory ligand PD-L1, which binds to PD-1 on T cells (and B7 molecules) which bind to CTLA-4. Cancer cell engagement with inhibitory ligands (against PD-1 and CTLA-4) prevents cytotoxic killing of cancer cells. **B** Activated T-cell against cancer cell. CD8^+^ T cells recognize tumor-associated antigens expressed on MHC class I on tumor cells (red spot) via the TCR, which results in cytotoxic killing of cancer cells via release of granzymes and perforins (cytokines). Inhibitory ligands (e.g. against PD-1 and CTLA-4) are blocked by immune checkpoint inhibitors (ICI), allowing active T cell function towards cancer cells. ICI drug classes are mentioned in squares, color depicting monoclonal antibodies against targeted receptors. Several associated mechanisms are involved (e.g. dendritic cells) which results in cytotoxic killing of cancer cells via release of granzymes and perforins (cytokines) leading to cancer cell death. CTLA-4, cytotoxic T lymphocyte-associated protein 4; PD-1, programmed cell death 1; PD-L1, PD-1 ligand. Created in part with Biorender

Immune checkpoint inhibitors (ICI) constitute a class of immunotherapy drugs that target the immune evasion by cancers cells used to avoid immune cell destruction and cell death (Fig. 1). Patients with dMMR primary tumors have a strong peri-tumoral immune reaction that is related to a favorable prognosis and to response to immune checkpoint inhibitors (ICI) [2, 14, 15], including pembrolizumab and nivolumab [16, 17]. Both pembrolizumab and nivolumab are programmed cell death protein 1 (PD-1) inhibitors and were approved by the US Food and Drug Administration as first line treatment of dMMR metastatic CRC [18]. Since then, ipilimumab, a cytotoxic T-lymphocyte-associated protein-4 (CTLA-4) inhibitor, has been approved in combination with nivolumab for use in metastatic CRC. Of note, a wide range of further drug-targets for immune checkpoint inhibition are being tested. The most important drugs used in ongoing neoadjuvant trials identified in this review and their action mechanisms are depicted in Fig. 1B. The ICI drugs are investigated with or without concomitant preoperative radiotherapy or chemoradiotherapy, with both short course and total neoadjuvant therapy as designed interventions.

**Fig. 2:**
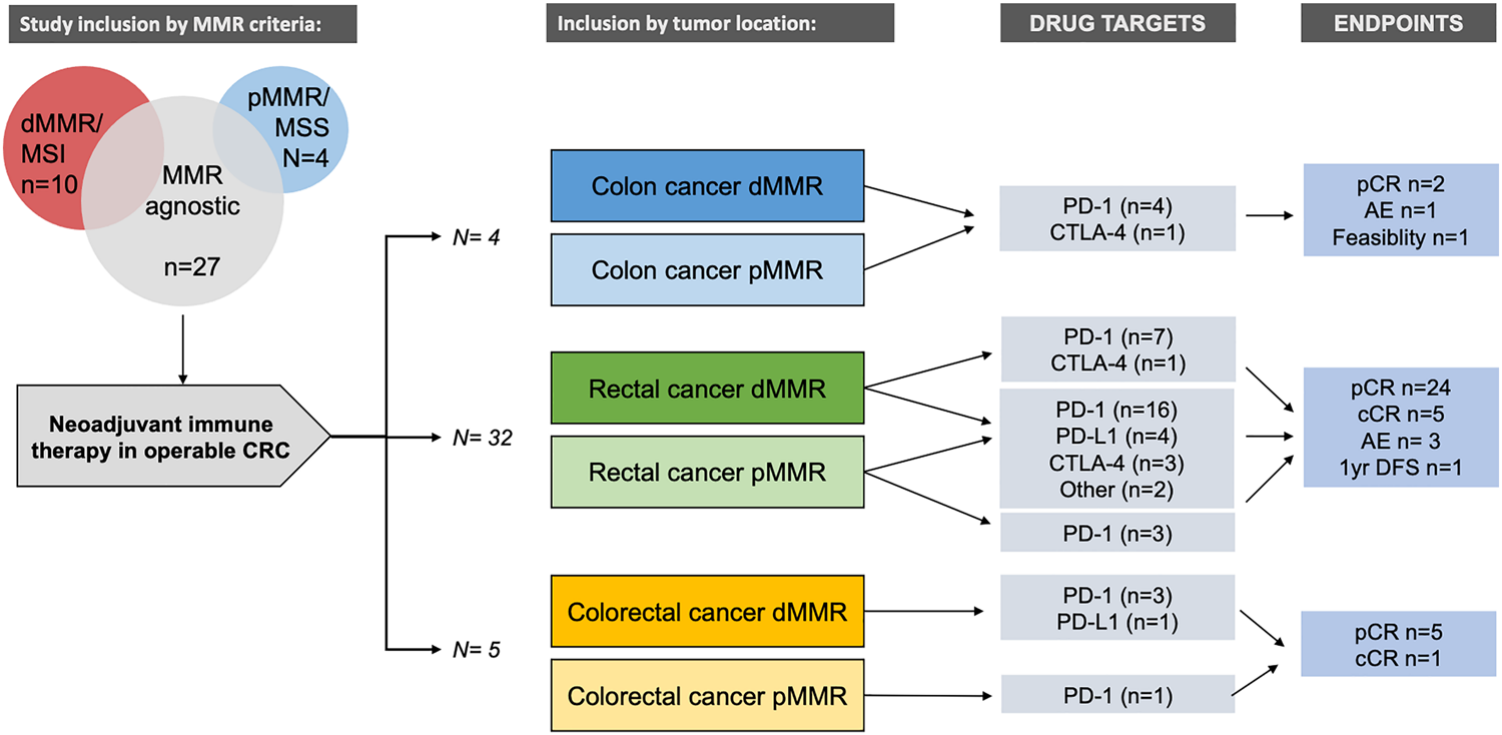
Trial overview with inclusion, tumor location, drug targets and endpoints. Numbers do not add up as there is overlap in some categories (e.g. 4 studies use both PD-1 and CTLA-4 inhibitors) and 3 studies report both pathological and clinical complete response (pCR and cCR) as the primary outcomes/study endpoints

Only a few trials are exclusively testing and selecting cancers with dMMR for inclusion, although the highest response rates to ICI in CRC are arguably demonstrated in relation to dMMR status [7, 8, 19, 20]. A pathological or clinical complete response is the most frequently used endpoint (Fig. 2), with a range of feasibility and efficacy measures investigated (Tables 1, 2; extended information is provided in Supplementary info). The neoadjuvant ICI treatment duration is scheduled for two and up to six cycles before intended surgery in the trials.

In metastatic CRC, the rate of dMMR is reported to less than five per cent. Less than three per cent of operable rectal cancers have dMMR [4, 21, 22]. The highest prevalence of dMMR cancers is found in the colon (about 20–25%), with highest predilection for right-sided colon cancers [23, 24]. Despite the much higher prevalence of dMMR in locally advanced colon cancer (MSI reported at 20–25%), ICI has not yet been approved for upfront treatment in primary stage I–III cancer of the colon. Similarly, while rather rare in rectal cancers (< 3% have MSI) the reported small series have shown remarkable effect, yet ICI is not approved for rectal cancers planned for surgical resection. Recently, the NICHE-1 study reported remarkable results from only two doses of neoadjuvant ICI in colon cancer before surgery, with 20 of 20 having some pathological response, 19 of 20 a major pathological response, and 60% (12 of 20) a complete pathological response in the dMMR group [8]. For rectal cancer, a study of dostarlimab (a PD-1 blocker) given to 12 patients with dMMR locally advanced rectal cancer [7] demonstrated a 100% clinical response rate at 12 months follow-up [7], assuring an organ-sparing strategy in all patients, although long-term data are awaited. The results from the NICHE-1 study was further corroborated by a phase II study, the PICC trial [6], investigating toripalimab with or without celecoxib in both colon and rectal cancer patients, reporting up to 88% pathological complete response. Hence, there seems to be a set of patients with early-stage, resectable CRC that clearly benefits from pre-operative immunotherapy.

#### Study types and trial design

The ongoing studies are early clinical trials (phase II studies), with only three phase III trials identified (Tables 1 and 2). The studies investigate ICI in a neoadjuvant setting either alone or, together with chemotherapy, chemoradiotherapy (CRT) or radiotherapy. Inclusion criteria are reported as patients with ECOG score 0–1 and age ≥ 18, and both sexes included. Notably, most studies do not discriminate specifically between MSI or dMMR status for inclusion (Fig. 2; Tables 1 and 2).

#### Neoadjuvant ICI trials in colon cancer

Four studies focus exclusively on ICI treatment for colon cancer. All studies target PD-1, but one study with the addition of CTLA-4 antibody together with the PD-1 antibody. Patients are scheduled to receive two to six cycles of ICI before surgery. All four studies include patients with both MSI/dMMR and MSS/pMMR tumors. Two of the studies gave ICI as monotherapy.

In the NICHE-1 study [8], celecoxib (a selective cox-2 inhibitor) was administered together with ICI. One study stands out by giving ICI and chemotherapy after stent-treatment for stenotic cancer with two cycles of camrelizumab and two cycles of CapeOX or three of FOLFOX, followed by surgery 2–3 weeks after the last round of chemotherapy. Another study in the colon group (JFSOL trial) has two treatment arms: one with ICI and six neoadjuvant cycles of chemotherapy (FOLFOX) and six adjuvant cycles of chemotherapy, and one arm with only chemotherapy both pre- and postoperatively. The primary outcome for two studies is response rate, either as clinical or pathological response evaluation. One study had as primary outcome adverse events, but secondary outcome included pathological response rate.

The NICHE-1 study on safety and efficacy of ICI therapy demonstrated remarkable response rates for dMMR cancers, but also response in 30% of the pMMR colon cancers [8]. In the follow up data, available as conference abstract [25], 30 patients with pMMR and 32 with dMMR tumors were evaluable for the efficacy analyses. At a median follow-up of 25 months (IQR 12–35 months), three patients (all non-responders) in the pMMR group had disease recurrence. In the 32 patients with dMMR tumors, pathologic response was observed in 100% of patients, with 31/32 (97%) having major pathological response. Pathological complete response was observed in 22 of 32 (69%, 95% CI 53–85%) patients. None of the patients in the dMMR cohort had disease recurrence [25]. In the continuation cohort the NICHE-2 study [26] have included 112 patients with dMMR colon cancer. After neoadjuvant ICI, three patients had postponed surgery due to adverse events and a pathological response was observed in 106 of 107 (99%) of evaluated patients.

The use of coxibs in combination with ICI was corroborated in the PICC trial [6]. This trial was based on dMMR or MSI as inclusion criteria, and 30 of the 36 patients had colon cancer (two had dual colon and rectal cancer). A clinical response was achieved in 95% of patients, with best response noted for toripalimab with celecoxib compared to monotherapy, and pathological complete response was highest in dual (88%) compared to monotherapy (65%). At a medium of 14.9 months follow up, all patients were alive and recurrence free [6].

#### Neoadjuvant ICI trials in rectal cancer

Most ongoing studies (Table 1) on neoadjuvant ICI are found in rectal cancer. The studies take advantage of a neoadjuvant treatment plan in locally advanced rectal cancer using chemoradiotherapy. Neoadjuvant ICI is added to the intended treatment. Hence, of the 32 studies on rectal cancer, 31 trials investigate immunotherapy added to intended neoadjuvant chemoradiotherapy treatment. Several of the studies include patients with pMMR cancers, despite the known poor response to ICI treatment in pMMR cancers. However, in theory, radiotherapy may induce immunogenicity, and hence provide an opportunity for ICI treatment in this setting [13, 27]. Twenty-three studies are agnostic for MMR/MSI status and include both tumor types. Several studies do not mention MSI/MMR status in inclusion or exclusion criteria. Six studies include MSI/dMMR tumors only, while three studies include exclusively MSS/pMMR tumors and one of the studies include MSS tumors positive for POLE mutations only. Pathological response rate is the most common reported primary outcome (Fig. 2).

All three phase III trials in neoadjuvant ICI are in patients with rectal cancer and are designed as multicenter, randomized, open label studies (Table 1). The largest trial (the STELLAR II study) aims to include 588 patients and allows for “watch and wait” as an option to responders. Therefore, both clinical and pathological complete response is used as a primary outcome. The second largest phase III study, the UNION trial, aims to include 213 patients investigating the PD-1 inhibitor camrelizumab in combination with radiochemotherapy using pathological complete response as the primary endpoint. The third phase III trial (NCT05215379) is a Chinese multicenter trial for patients with ultralow rectal cancers that are pMMR/MSS and a desire to preserve the anus who will be offered sintilimab (xintilimab) together with neoadjuvant radiochemotherapy. The study follows the preliminary results from a phase II trial, previously reported in abstract form [28].

The first study on neoadjuvant ICI in rectal cancer was published in 2021 [29], including 30 patients with clinical stage T3-4 N0 M0 or T1-4 N + M0 rectal cancer. Patients received short course radiotherapy followed by chemotherapy with CAPOX plus camrelizumab, a PD-1 inhibitor. Three patients were excluded as they were unable to complete chemotherapy. Of the 27 patients that completed treatment, only one had dMMR tumor and had a pathological complete response. Of the 26 others, 12 (46.2%) had a pathological complete response. All over, the pathological complete response rate was 48.1% (13/27) [29].

A study from the Memorial Sloan Kettering Cancer Center [7] reported remarkable response rates in patients with dMMR rectal cancer treated with dostarlimab. In the preliminary results, 12 patients had completed treatment with dostarlimab, and all patients had clinical complete response. The protocol planned for ICI to be followed by standard chemoradiotherapy and surgery. However, after inclusion of 12 patients with 100% clinical complete response after only neoadjuvant ICI (at 6–25 months of follow-up), none of the 12 patients were given chemoradiotherapy nor had surgery, nor developed recurrence. Planned inclusion is a total of 53 patients.

One open-label, phase 2, randomized clinical trial (NRG-GI002) [30] reported initial results from the pembrolizumab arm of a phase II randomized clinical trial on total neoadjuvant chemotherapy (TNT) in rectal cancer. Patients with distal rectal cancer (< 5 cm from anal verge, cT3-4, any N), with bulky disease (cT4 or tumor within 3 mm of mesorectal fascia), at high risk for metastatic disease (cN2) or that were not candidates for sphincter-sparing surgery was eligible. Of 185 patients included, 90 were randomized to the intervention arm with FOLFOX for 4 months with pembrolizumab before surgery. The surrogate endpoint in this trial was determined using the neoadjuvant rectal (NAR) score, which has questionable informative value over T- and N-stage across studies [31–33]. In the NRG-GI002 trial [30], the investigators found no improvement in the NAR score between the two groups leading the investigators to conclude that pembrolizumab added to FOLFOX in a neoadjuvant setting using TNT is not supported. The clinical and pathological complete response rates were 13.9% and 31.9% for pembrolizumab and 13.6% and 29.4% in the chemotherapy alone group [30]. However, both the choice of endpoint and the MMR agnostic approach to the study should be considered in the interpretation of the findings.

The Voltage trial published abstract at ASCO 2020 [34], reported promising pathological complete response rates in both MSI and MSS rectal patients, with 60% and 30%, respectively, after treatment with nivolumab in addition to chemoradiotherapy.

#### Neoadjuvant trials of ICI in combined colon and rectal cancers

Six studies (Table 2) include both colon and rectal cancer. The PICC-trial reported short-term follow-up [6] with very high response rates (88%), but long-term follow-up with survival data is pending. For studies combining colon and rectal cancers, several drug combinations are investigated (Table 1). Neoadjuvant duration is scheduled up to six cycles before surgery. Four of the studies only included patients with MSI/dMMR and in one trial only patients with MSS/pMMR are included. One of the studies includes an “watch-and wait” option for patients with rectal cancer who have clinical and radiological complete response. The study investigates ICI in locally advanced colon cancer (defined as T3/T4 tumor with extramural extension over 5 mm) and locally advanced rectal cancer (clinical T3/T4). Patients without response to ICI are offered rescue chemotherapy and rescue chemoradiotherapy, respectively. For all five studies combining colon and rectum, the primary outcome is reported as complete response, either as ‘pathological’ (in surgical specimen) or ‘clinical’ in the “watch and wait” arm.

#### Trials considering ‘watch and wait’ after response evaluation

Six studies include an option of ‘watch and wait’ for patients with rectal cancer and clinical complete response (Table 1). Only one of these (the Basket trial) is upfront designated as a ‘watch and wait’ study, with 1 year disease free survival as primary outcome. In the Basket trial, ICI is given in monotherapy without any chemotherapy or radiotherapy (expected to complete in 2024).

Five studies (NCT04558684, NCT04443543, NCT05484024, NCT04165772, NCT04715633; Table 1) add ICI together with chemoradiotherapy or total neoadjuvant treatment for rectal cancer. For patients with a clinical complete response, watch and wait is offered as an option or alternative. Following on the STELLAR trial of TNT in locally advanced rectal cancer [35], the Chinese STELLAR II trial investigates the addition of ICI in one arm. The STELLAR II Study is one of the largest trials and has planned accrual of 588 patients with the primary outcome as pathological or clinical response rate. In this trial, the patients are treated with short course radiotherapy and chemotherapy with CAPOX or mFOLFOX in the standard group and, in the experimental group the PD-1 inhibitor sintilimab is added to the same regimen.

#### Modulating immunogenicity in colorectal cancer

Early-stage CRC have higher CD8 + T-cell infiltration compared to metastatic disease, which suggest that primary tumors are more immune active than advanced or metastatic cancers [11, 36, 37]. Thus, one may perceive that early-stage CRC may have a better response to ICI than metastatic cancer, regardless of MSI/MSS status. However, proficient MMR or MSS cancers have shown poorer response to immunotherapy overall compared to dMMR [25, 38–40]. Conversely, dMMR rectal cancers seems to have a better tumor response to chemoradiation than chemotherapy alone [41]. Thus, there is an interest to investigate how ICI can further enhance tumor response in both dMMR and pMMR cancers [13, 38, 42, 43].

In some rectal cancers, it is believed that radiotherapy and chemotherapy have an immunomodulating effect on tumor cells [38, 44], and thereby increasing the probability of response to ICI even in MSS/pMMR tumors. In experimental study in mice, radiotherapy given as 10 Gy × 5 increased the expression of PD-L1 in tumor cells [45]. The study was conducted to enhance the therapeutic efficacy of radiotherapy in an experimental setting. However, as reviewed extensively elsewhere, it also supports an increasing line of data that suggests radiotherapy can be immunomodulating in pMMR cancers [13, 38, 46].

A study from Japan showed that neoadjuvant chemoradiotherapy on rectal cancer increased the CD8 + tumor infiltrating lymphocytes compared to patients not given chemoradiotherapy [47], suggesting that anti-tumor immunity was enhanced by chemoradiotherapy. Whether an immunomodulating strategy holds true, remains to be shown. Further basic science and translational data are needed to better understand how an immunomodulating effect could be initiated in pMMR cancers.

#### The role of MMR status in ICI selection

The studies on neoadjuvant ICI for colorectal cancer show that ICI has best effect in MSI/dMMR tumors. However, an effect on pathological response rate is also observed in about 30% of MSS/pMMR tumors [6, 8, 34, 48]. For locally advanced rectal cancer, neoadjuvant chemoradiotherapy is given prior to surgery to decrease the risk of local recurrence for selected patients. In some patients, the treatment results in a pathological complete response [49, 50]. Hence, an increased interest in a “watch and wait” strategy for patients with a clinical complete response after neoadjuvant therapy has emerged, to allow for an organ-sparing management approach [51, 52].

In rectal cancer, ICI is thus investigated (for the most part) as an additional add-on to ongoing investigations of pre-operative chemo-radiotherapy regimens for locally advanced rectal cancer. The VOLTAGE-trial [48] presented data from 38 patients with MSS CRC with two times the rate of pathological complete response (67% vs. 30%) in a subgroup of patients given neoadjuvant chemoradiotherapy with nivolumab, compared to the whole group of MSS rectal cancer. In a further single-arm, phase II trial, neoadjuvant ICI was combined with short-course radiotherapy before surgery [29]. In the trial, the use of chemotherapy with CAPOX and camrelizumab showed pathological complete response rates of 48.1% for all included (13 of 27), with one patient having dMMR and 12 of 26 (46.2%) having pMMR cancers [29]. The study corroborates the response achieved in pMMR cancers. However, the effect from chemotherapy and ICI treatment is difficult to differentiate as it is difficult to prove causality from the non-randomized data. Data on survival is currently lacking.

A further retrospective study [53] of 33 colorectal patients with clinical stage T3N0-2M0 and MSI or dMMR tumor was treated with PD-1 inhibitor for 4–10 cycles (median 6), and had a pathological response rate of 100%, with 22/29 (76%) achieving pathological complete response. Three of the patients had clinical complete response and were offered a ‘watch and wait’ strategy. Notably, eight patients had either failure or, no response, to neoadjuvant chemoradiotherapy or chemotherapy but had pathological response to ICI treatment.

#### Other criteria suggesting effect of neoadjuvant ICI

The current understanding is that dMMR/MSI, together with tumor mutational burden (TMB) and PD-1/PD-L1 expression, plays a role as a predictive biomarker for immunotherapy selection [19]. The TMB in MSI tumors is suggested to differ according to CMS subtypes, with MSI tumors from CMS1 being most immunogenic [54]. This could suggest different susceptibility to ICI in MSI tumors and those with a non-MSI related high TMB and (Supplementary Fig. 1) thereby differences in clinical response [55–58]. So far, this has not been shown in the studies on ICI in primary CRC. In a large untreated cohort of 738 rectal cancers exploring genomic and transcriptomics patterns of neoadjuvant response, RNA-sequencing estimates of immune infiltration identified a subset of pMMR cancers that were deemed immune hot tumors with increased response and prolonged disease-free survival [59]. Hence, further translational research into the mechanisms that illicit an immune response or effect of ICI is warranted.

Most studies of neoadjuvant ICI concern the use of PD-1 inhibitors [60] (Fig. 2). As PD-L1 immunohistochemistry testing is done for other tumors [16, 61], selection based on PD-L1 staining would seem intuitive [62]. However, there are several concerns about the different assays, methods of evaluation in different tumor types, scoring criteria for PD-L1, and the impact of the results on therapeutic decisions [63]. Hence, a uniform and standard approach using PDL-1 staining is not available, despite a strong correlation of PD-L1 to MSI and immune-cell infiltration in early-stage CRC [11].

In CRC, tumors with POLE mutation have high levels of immune cell infiltration with CD3 + and CD8 + T-cells and NK cells comparable to MSI tumors [64, 65]. In a case presentation, complete clinical and radiological response for an MSS POLE positive metastatic CRC patient was reported [66]. Hence, this rare mutation could serve as a selection for ICI treatment in some patients (Supplementary Fig. 1). Also, patients with NTRK fusion have a high TMB and high MSI, and an enrichment for POLE/POLD1 mutations, suggesting these to be a subset for ICI treatment as well [67].

#### Need for larger studies with improved study endpoints

The ongoing trials (Tables 1, 2) include a limited number of patients. Based on the results published so far, the possibility for organ-sparing treatment of MSI/dMMR colon cancer and rectal cancer after neoadjuvant ICI treatment could be considered. However, the lack of phase III studies in which ICI is compared to a standard neoadjuvant regimen leaves this question largely unanswered. Also, the surveillance of colon cancer has several clinical and methodological problems compared to rectal cancer, as mentioned below.

A further concern from ICI given in the preoperative setting, is the durability of response, particularly for those in “watch and wait” surveillance. For durability assessment, only extrapolated data from trials in metastatic CRC can be considered. For metastatic CRC [68], the median progression free survival for metastatic MSI/dMMR CRC was 16.5 months on pembrolizumab compared to 8.2 month on chemotherapy. However, even if the response to ICI was longer than for chemotherapy alone, it was not durable. Whether the response in early-stage CRC is more durable and even results in a lasting (that is, curative) response, is currently unknown. The comparator intervention is survival data from upfront surgery in early-stage CRC, which is known to have a high curative rate (albeit not perfect). Hence, the expected efficacy of ICI must be higher in a curative setting for early-stage colon and rectal cancers. In the case of a lower efficiency in ICI compared to conventional surgery (and adjuvant) for colon cancer, one must factor in the baseline recurrence and metastasis risk in both scenarios.

For locally advanced rectal cancer, outcome needs to balance the recurrence-risk as well as the rate of organ-sparing achieved (i.e. no stoma and no loss of function in a subset of patients) and, patients’ willingness of a risk–benefit balance between the oncological outcomes and quality of life. Also, one needs to be reassured that, if recurrence should occur, that recurrent disease is of a similar tumorbiological type that is amenable to salvage surgery and treatment and, not converted to a biologically more aggressive, non-curative subtype.

Currently, studies in colon cancer have shown the highest rates of complete response for dMMR patients after neoadjuvant ICI. However, no studies propose ‘watch and wait’ for patients with colon cancer and clinical complete response. This is largely due to the much more difficult access to the colon for surveillance (i.e. full colonoscopy), compared to surveillance of the rectum that can be more readily assessed by both cross-sectional imaging, endoscopy (by anorectoscopy), biopsy and, digital rectal exam.

### Future directions

The reported response rates in dMMR cancers points to a clear role for ICI in future treatment of colorectal cancer beyond the metastatic setting. However, it is not clear what regimen, alone or in combination, should be preferred for which patients. For pMMR [38], further studies and biomarkers of potential response need to be developed in order to offer this group ICI treatment which translates into response [3, 40, 69]. Further understanding, reporting and management of toxicities is important [70], particular as most ongoing neoadjuvant studies only investigates short durations of ICI treatment.

A ‘watch and wait’ strategy is feasible in rectal cancer due to several modes of surveillance; imaging with MRI and/or PET scans, endoscopic, biopsy and digital rectal exploration al allows for assessment of the lower rectum to gauge and monitor response closely. As surgery for locally advanced or low rectal cancers may be mutilating, a high or even complete response may allow for an organ-sparing approach, as already investigated by conventional chemo-radiotherapy [71]. Of note, the response criteria are not uniform across centers and studies in this regard [72], with risk for regrowth and distant metastasis influenced by inclusion criteria across cohorts [73]. However, with the current knowledge of the lower chemo response rates in rectal cancers with MSI/dMMR and the data on ICI response, suggests a personalized approach with appropriate molecular testing in rectal cancer is warranted [74], possibly foregoing or refraining from any standard chemo-radiation in ICI responders.

A ‘watch and wait’ approach is not readily available in the same fashion for colon cancer. One major obstacle is the response evaluation by imaging, which is less well-described for colon cancer compared to rectal cancer. Also, MRI scanning is not currently a viable option for colon cancer. Hence, neoadjuvant studies for dMMR colon cancers have so far involved completion of the planned surgery, even if the highest response rates are seen in colonic cancers. Improved surveillance strategies are needed, e.g. with robust and validated response criteria in imaging studies or sequential use of liquid biopsies to follow response. Data suggest that imaging biomarkers may soon be available [75], but need to be accurate, robust and, valid for the clinical situation. Furthermore, circulating DNA may become a valid tool for surveillance, but translational studies of liquid biopsy use in the neoadjuvant ICI setting for colon and rectal cancer needs to be better defined [76].

Finally, hard endpoints including overall survival, disease-free survival and, recurrence risk over time needs to be investigated. Also, one needs to be reassured that relapse from initial response does not lead to a biology shift towards more aggressive disease in patients treated with ICI and not undergoing intended surgery. After all, a vast majority of stage I–III CRC is currently cured by surgery alone (with selective use of adjuvant therapy) with very good survival, and hence a pure immunotherapy approach followed by a non-operative, organ-sparing strategy should be at least as oncological safe as modern surgery for stage I–III colorectal cancer surgery.

## Conclusion

Early clinical data have shown a profound effect of neoadjuvant ICI in resectable early-stage, colon and rectal cancer. However, neoadjuvant therapy with ICI is not yet established treatment and the clinical importance is yet to be determined. Most studies are MMR/MSI agnostic. Three phase III studies investigate ICI added to chemoradiotherapy for rectal cancer. Six studies in rectal cancer include “watch and wait” as an option for organ- and function-sparing approach in clinical complete responders. To move forward, further studies including translational endpoints and larger phase III studies are required.

## Supplementary Information

Below is the link to the electronic supplementary material.

Supplementary file1 (DOCX 37 KB)

Supplementary file2 (DOCX 325 KB)
