# Supplementary material for: Clinical trials of neoadjuvant immune checkpoint inhibitors for early-stage operable colon and rectal cancer
Source: Cancer Immunol Immunother. 2023 Aug 1;72(10):3135–47. doi: 10.1007/s00262-023-03480-w (PMC10491705; doi:10.1007/s00262-023-03480-w)
Supplement: Supplementary file 1 — Supplementary file1 (DOCX 37 KB) [file 262_2023_3480_MOESM1_ESM.docx]

| **Neoadjuvant immunecheckpoint inhibitor trials in colon cancer** | | | | | | | | | | | | | |
| --- | --- | --- | --- | --- | --- | --- | --- | --- | --- | --- | --- | --- | --- |
| **NCT #/EudraCT** | **Drug** | **Study start** | **Ca completion** | **Target** | **Mono/ double therapy** | **N** | **Phase** | **Stage** | **Cancer location** | **MSI/MSS** | **Watch and wait** | **Outcome** | **Results** |
| NCT03985891  JSFOL | JS001/ Toripalimab | 16.12.20 | 01.06.25 | PD-1 | Mono (+ Chemo) | 40 | I/II | III | C | Both | No | pCR, rCR | No |
| NCT05202314  (NACSOC-02) | Camrelizumab | 12.12.21 | 30.12.23 | PD-1 | Mono (+chemo) | 20 | I/II | II-IV | C | Both | No | pCR | No |
| NCT03026140  NICHE  NICHE-2 | Nivolumab, Ipilimumab | 29.3.17 | 06.21 | PD-1, CTLA-4 | Double | 60  130 | II | I-III | C | Both  MSI | No | AE  3yr DFS | Yes  Yes |
| NCT04231526 | Pembrolizumab | 20.05.20 | 19.03.25 | PD-1 | Mono | 46 | II | II/III | C | Both | No | Feasibility | No |
| **4 studies** | | | | | | | | | | | | | |
| **Neoadjuvant immunecheckpoint inhibitor trials in rectal cancer** | | | | | | | | | | | | | |
| **NCT #/EudraCT** | **Drug** | **Study start** | **Ca completion** | **Target** | **Mono/ double therapy** | **N** | **Phase** | **Stage** | **Cancer location** | **MSI/MSS** | **Watch and wait** | **Outcome** | **Results** |
| NCT03854799  AVANA | Avelumab | 01.04.19 | 12.23 | PD-L1 | Mono (+ CRT) | 101 | II | III | R | Both | No | pCR | No |
| NCT04357587 | Pembrolizumab | 06.08.20 | 12.22 | PD 1 | Mono (+CRT) | 10 | I | III-IV | R | MSI/dMMR | No | AE, CSOT | No |
| NCT03127007 (R-IMMUNE) | Atezolizumab | 27.4.17 | 01.23 | PD-L1 | Mono (+CRT) | 54 | I/II | III | R | Both | No | AE, pCR | No |
| NCT04017455  TARZAN | Atezolizumab  Bevacizumab | 22.10.19 | 20.08.23 | PD-L1  VEGF | Mono (+RT) | 38 | II | I-III | R | Both | No | cCR | No |
| NCT04109755  PEMREC | Pembrolizumab | 02.06.20 | 06.22/03.28 | PD-1 | Mono  (+RT) | 25 | II | III | R | MSS | No | TRR | No |
| NCT04130854  INNATE | AOX005M/ Sotigalimab | 24.4.20 | 01.11.23 | antiCD40 | Mono (+CRT) | 58 | II | II-III | R | Both | No | pCR | No |
| NCT04503694  Regina trial | Nivolumab | 25.03.21 | 09.23 | PD-1 | Mono (VEGF + radio) | 60 | II | II/III | R | Both | No | pCR | No |
| NCT04518280  TORCH | Toripalimab | 01.05.21 | 31.12.23 | PD-1 | Mono (+CRT) | 130 | II | II/III | R | Both | No | pCR | No |
| NCT04558684 | Camrelizumab | 01.11.19 | 30.12.23 | PD-1 | Mono(+CRT) | 30 | I/II | II/III | R | Both | Consider wathch and wait | cCR, DFS | No |
| NCT05176964 | Tislelizumab | 31.12.21 | 31.12.24 | PD - 1 | Mono (+CRT) | 50 | II | II/III | R | Both | No | pCR | No |
| NCT 04621370  PRIME-RT | Durvalumab | 07.12.20 | 30.06.25 | PD-L1 | Mono (+ CRT) | 48 | II | III | R | Both | No | pCR | No |
| NCT04231552 | Camerelizumab | 10.11.19 | 30.09.23 | PD-1 | Mono (+CRT) | 30 | I/II | III | R | Both | No | pCR | Yes |
| NCT04643041  (BASKET) | ? | 01.01.21 | 31.12.26 | PD-1 | Mono | 47 | Not applicable | I-III | R | MSI/dMMR | Watch and wait | 1-yr DFS | No |
| NCT 03921684 | Nivolumab | April-19 | April - 21 | PD1 | Mono (+CRT) | 29 | II | III | R | Both | No | pCR | No |
| NCT04906044  STARS-RC03 | Tislelizumab | 1.6.21 | 1.6.28 | PD-1 | Mono (+CRT) | 30 | I | I-III | R | Both | No | AE | No |
| NCT02921256  (NRG-GI002) | Pembrolizumab | 12.10.16 | 21.3.23 | PD-1 | Mono (+CRT) | 362 | II | II/III | R | Both | No | Imrove Neadjuvant rectal cancer score | Yes |
| NCT04443543 | Tislelizumabb | 22.06.20 | 30.06.26 | PD-1 | Mono (+CRT) | 222 | II | II/III | R | Both, but only MSI given immunth | Watch and wait | cCR | No |
| NCT04751370 | Nivolumab, Ipilimumab | 13.5.21 | 30.9.22 | PD-I/ CTLA-4 | Double (+RT) | 31 | II | II/III | R | MSI/dMMR | No | pCR | No |
| NCT03503630 | Compound  2055269 | 20.7.18 | 2.1.24 | ? PD-1? | Mono (+CRT) | 44 | II | II/III | R | Both | No | pCR | No |
| NCT02688712  ExIST | Galunisertib | 06.16 | Des-21 | TGFbeta type 1 receptor inhibitor | Mono (+CRT) | 50 | II | II-IV | R | Both | No | pCR | No |
| NCT02948348  VOLTAGE | Nivolumab, Ipilimumab | 10.16 | 12.21 | PD-1  CTLA-4 | Mono (+ CRT)  Double (+CRT) | 90 | I/II | III/IV (one arm with resectable mCRC) | R | Both | No | pCR | Yes – presentet at ASCO |
| NCT04928807  (UNION) | Camrelizumab | 20.7.21 | 20.7.22 | PD-1 | Mono (+CRT) | 213 | III | III | R | Both | No | pCR | No |
| NCT04663763 | Sintilimab | 1.12.20 | 1.12.22 | PD-1 | Mono (+CRT) | 40 | II | II/III | R | Both | No | pCR | No |
| NCT04411537 | PD-1-antibody | 01.07.20 | 30.04.22 | PD-1 | Mono (+CRT) | 50 | II | II/III | R | MSS/pMMR | No | pCR | No |
| NCT04411524 | PD-1-antibody | 01.07.20 | 30,04.22 | PD-1 | Mono  (+CRT) | 50 | II | II/III | R | MSI/dMMR | No | pCR,DFS | No |
| NCT05507112 | Tislelizumab | 20.08.22 | 01.07.24 | PD-1 | Mono (+CRT) | 100 | II | III | R | MSI/dMMR | No | pCR | No |
| NCT05484024  (STELLAR II) | Sintilimab (Tyvyt) | 06.08.22 | 31.07.28 | PD-1 | Mono (+CRT) | 588 | II/III | III | R | Both | Watch and Wait optional if cCR | pCR/cCR | No |
| NCT05479240 | Tislelizumab | 01.09.22 | 01.09.26 | PD-1 | Mono (+CRT) | 96 | II | II/III | R | Both | No | pCR | No |
| NCT05215379 | Xintilimab (Tyvyt) | 1.8.22 | 1.4.25 | PD-1 | Mono (+CRT) | 180 | II/III | II/III | R | MSS | No | cCR at 6 mth (WW) | No |
| NCT04124601/  2019-003865-17  CHINOREC | Ipilimumab  Nivolumab | 01.06.20 | 31.12.23 | CTLA-4  PD-1 | Double (+CRT) | 80 | II | II/III | R | Both | No | Safety, feasibilty | No |
| NCT04165772 | Dostarlimab | 11.12.19 | 30.11.23 | PD- 1 | Mono (+CRT) | 51 | II | II/III | R | MSI/dMMR | Watch and wait if cCR | pCR/cCR | Yes |
| NCT04293419 | Durvalumab | 18.12.19 | 18.11.24 | PD-L1 | MONO (+CRT) | 58 | II | III | R | Both | No | pCR | No |
| **32 studies** | | | | | | | | | | | | | |
| **Neoadjuvant immunecheckpoint inhibitor trials in colon cancer and rectal cancer** | | | | | | | | | | | | | |
| **NCT #/EudraCT** | **Drug** | **Study start** | **Ca completion** | **Target** | **Mono/ double therapy** | **N** | **Phase** | **Stage** | **Cancer location** | **MSI/MSS** | **Watch and wait** | **Outcome** | **Results** |
| NCT03926338 | Toripalimab | 10.05.19 | 05.21 | PD-1 | Mono | 100 | I/II | I-III | C+R | MSI/dMMR | No | pCR | Yes |
| NCT04715633 | Camrelizumab  Apatinib | 01.12.20 | 31.12.22 | PD-1  VEGF | Double (+CRT or chemo only) | 52 | II | III | C+R | MSI/dMMR | For ca recti, Watch and Wait if cCR | cCR/pCR | No |
| NCT04895137  BASKETII | PD-1 monoclonal antibody | 1.05.21 | 05.22 | PD-1 | Mono(+chemo) | 42 | II | III | C+R | MSS/pMMR | No | pCR | No |
| NCT05371197 | Envafolimab | 25.05.22 | 30.06.24 | PD-L1 | Mono | 26 | II | II/III | C+R | MSI/dMMR | No | pCR | No |
| NCT05197322  NEOPRISM-CRC | Pembrolizumab | Juli-22 | Sept-27 | PD-1 | Mono | 32 | II | II/III | C (+evt R) | MSI/dMMR | No | pCR | No |
| **5 studies** | | | | | | | | | | | | | |

Abbreviations:

CRT = Chemoradiotherapy

RT = Radiotherapy

n.s. denotes “not specified”.

pCR = pathological complete response

AE = adverse events

CSOT = completed surgical or oncological treatment

cCR = clinical complete response

TRR = Tumor regression rate

DFS = disease free survival
