# Supplementary figures and images for: Clinical trials of neoadjuvant immune checkpoint inhibitors for early-stage operable colon and rectal cancer
Source: Cancer Immunol Immunother. 2023 Aug 1;72(10):3135–47. doi: 10.1007/s00262-023-03480-w (PMC10491705; doi:10.1007/s00262-023-03480-w)

For Supplementary info

**Figure 1. Search strategy and results.**


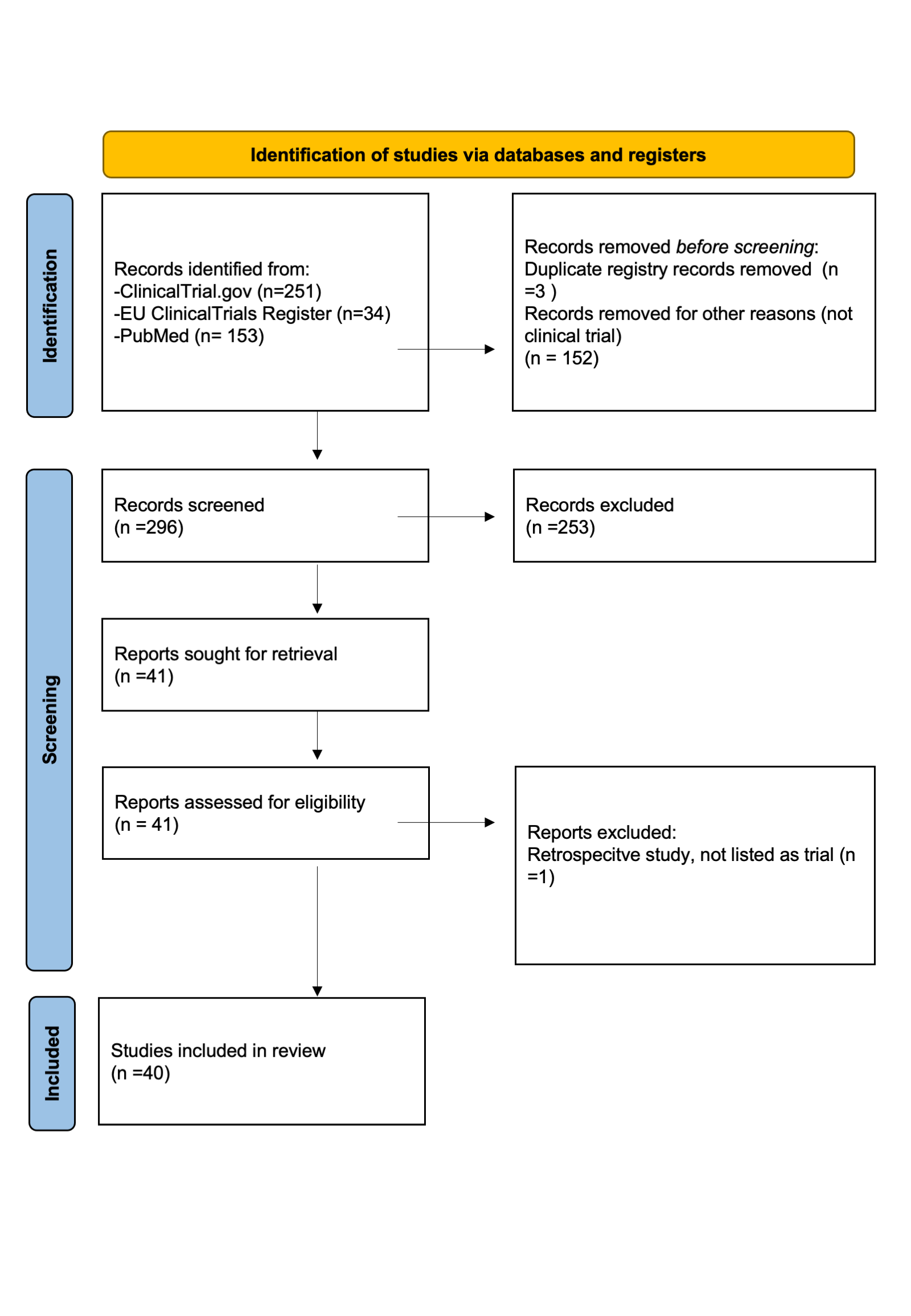

Supplement: Supplementary file 2 — Supplementary file2 (DOCX 325 KB) [file 262_2023_3480_MOESM2_ESM.docx]
